# ESR Method in Monitoring of Nanoparticle Endocytosis in Cancer Cells

**DOI:** 10.3390/ijms21124388

**Published:** 2020-06-19

**Authors:** Ryszard Krzyminiewski, Bernadeta Dobosz, Bart Krist, Grzegorz Schroeder, Joanna Kurczewska, Hans A.R. Bluyssen

**Affiliations:** 1 Medical Physics and Radiospectroscopy Division, Faculty of Physics, Adam Mickiewicz University, Uniwersytetu Poznanskiego 2, 61-614 Poznan, Poland; rku@amu.edu.pl; 2 Department of Human Molecular Genetics, Institute of Molecular Biology and Biotechnology, Faculty of Biology, Adam Mickiewicz University, Uniwersytetu Poznanskiego 6, 61-614 Poznan, Poland; bartkrist@gmail.com (B.K.); h.bluyss@amu.edu.pl (H.A.R.B.); 3 Faculty of Chemistry, Adam Mickiewicz University, Uniwersytetu Poznanskiego 8, 61-614 Poznan, Poland; schroede@amu.edu.pl (G.S.); asiaw@amu.edu.pl (J.K.)

**Keywords:** electron spin resonance, magnetic nanoparticles, TEMPOL spin label, breast cancer cells

## Abstract

Magnetic nanoparticles are extensively studied for their use in diagnostics and medical therapy. The behavior of nanoparticles after adding them to cell culture is an essential factor (i.e., whether they attach to a cell membrane or penetrate the membrane and enter into the cell). The present studies aimed to demonstrate the application of electron spin resonance (ESR) as a suitable technique for monitoring of nanoparticles entering into cells during the endocytosis process. The model nanoparticles were composed of magnetite iron (II, III) oxide core functionalized with organic unit containing nitroxide radical 4-hydroxy-TEMPO (TEMPOL). The research studies included breast cancer cells, as well as model yeast and human microvascular endothelial cells. The results confirmed that the ESR method is suitable for studying the endocytosis process of nanoparticles in the selected cells. It also allows for direct monitoring of radical cellular processes.

## 1. Introduction

Nanomaterials are often a research object for their use in diagnostics and medical therapy [1,2,3,4,5,6]. Iron (II, III) oxide nanoparticles as potential materials for use in medical imaging [7,8], magnetic hyperthermia [9,10,11], and drug delivery carriers [12,13] are of great interest.

Iron oxide nanomaterials are characterized using different methods, including transmission electron microscopy (TEM), X-ray diffraction (XRD), superconducting quantum interference device (SQUID), UV-vis spectroscopy, and dynamic light scattering (DLS) [14,15]. The electron spin resonance (ESR) method has previously been used for the description of the physical properties of liposomes [16,17], polymer nanoparticles [18,19,20], or graphene [21]. Indeed, ESR can be a useful method for the characterization of iron oxide nanomaterials [22,23,24,25].

The application of nanomaterials as drug delivery carriers requires careful analysis of cellular (and intracellular) interactions between nanomaterials and biological systems. Initially, the studies are carried out using cell lines [26,27], followed by tests on animals [28,29,30]. A fundamental issue considered in the application of new potential drug carriers based on nanomaterials is their ability to penetrate the cell membrane. [31,32,33]. The successful cellular uptake of nanomaterials depends on drug carrier size. Uptake occurs through endocytosis (nanoparticles from several tens to hundreds of nanometers) or direct permeation (nanoparticles smaller than 50 nm; 10 nm are more preferred) [34]. Endocytosis is a process in which membrane invagination leads to the formation of intracellular carrier vesicles that allow the capture of particles or fluids from the extracellular compartment [35]. The number of nanoparticles entering a cell and the ability of process monitoring is crucial for the practical application of nanodrugs in medical therapy. Currently, the process is monitored microscopically based on fluorescently labeled molecules. The presence of labeled molecules inside a cell causes fluorescence of this cell, and its intensity depends on the concentration of labeled molecules inside the cell [33,36]. The method’s weakness is that it gives information about the label attached, instead of the nanoparticle itself. Therefore, the number of nanoparticles that entered the cell cannot be determined. Nanoparticles functionalized with free radicals (spin labels) can be characterized by ESR, giving detailed information about the magnetic core and attached radicals at the surface [22,24,25]. The method provides data about the number of nanoparticles that penetrated into the cell and thus about the amount of drug delivered. Additionally, spin labels are used as scavengers of reactive oxygen forms in inflammation or cancer [3,4,37]. Therefore, ESR might be a more preferred method for iron oxide nanoparticle monitoring compared to microscopical observation.

The objective of this paper was to demonstrate the application of electron spin resonance as a suitable method to study interactions of functionalized magnetic nanoparticles with various types of cells, including cancer cells.

## 2. Results and Discussion

**Figure 1:**
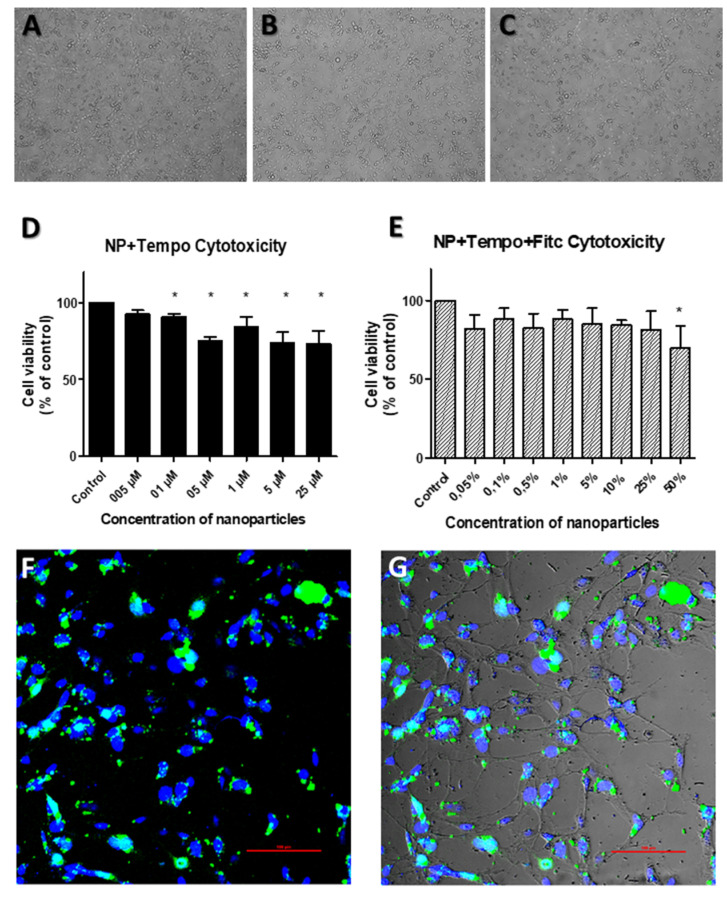
Functionalized nanoparticles do not exert cytotoxicity and enter the cells. (**A**–C): HMEC morphology is not changed by the addition of nanoparticles without (**B**) or with magnet (**C**), compared to nontreated control cells (**A**). Bright field images; magnification = 10x. (**D**–**E**): Cell viability as percentage of control for (**D**) nanoparticles with TEMPO spin label and (**E**) nanoparticles with TEMPO spin label and FITC; measured by EZ4U proliferation and cytotoxicity assay. Error bars represent mean + SD. * = *p* < 0.05 (one-way ANOVA). (**F**–**G**): Confocal fluorescent imaging of FITC labeled nanoparticles in HMEC cells; (**F**) only green and blue channels, (**G**) fluorescent channels + bright field. Magnification = 100x.

An important requirement for new drug delivery carriers is their nontoxicity. Therefore, initial studies concerned cytotoxicity of the nanoparticles on cells. Representative results for HMEC (human microvascular endothelial cells) are shown in Figure 1 and in Table 1, Table 2, Table 3 and Table 4. Although a significant decrease in cell viability is observed, a cell viability above 80% is maintained under all but the highest concentrations tested (25 µM and 50% of particle suspension). This suggests only a mild cytotoxic effect of the particles on the tested cells. On the other hand, they can enter a cell confirmed by confocal microscope pictures. As the nanoparticles satisfied the necessary condition, further analysis was carried out by the ESR method.

### 2.1. Effect of Temperature

**Figure 2:**
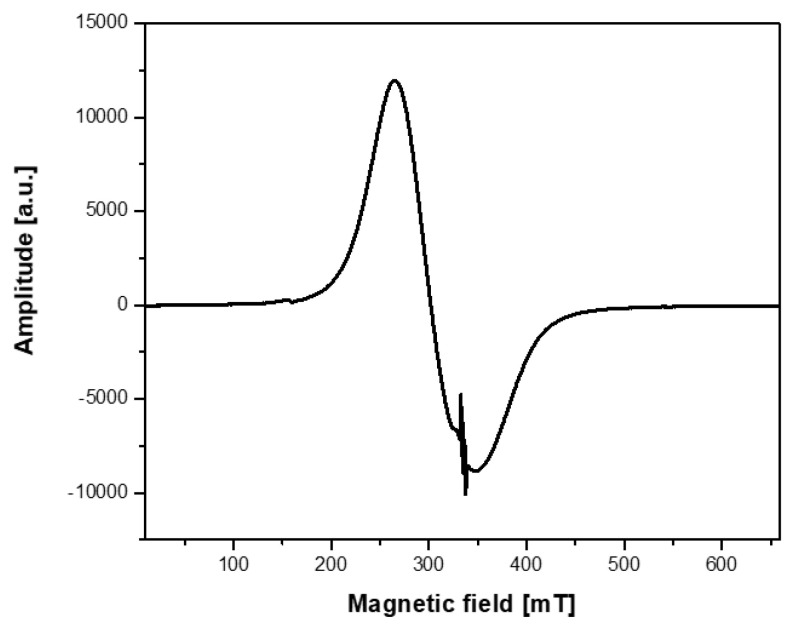
ESR spectrum of the Fe_3_O_4_@SiO_2_@SiNHDOX@Dextran-TEMPOL nanoparticles recorded in a magnetic sweep range of 650 mT at 293 K.

**Figure 3:**
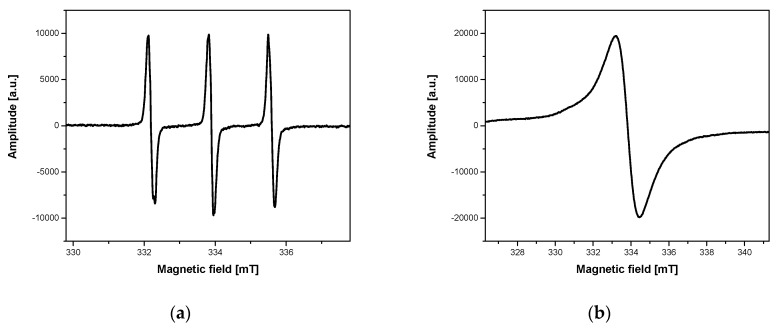

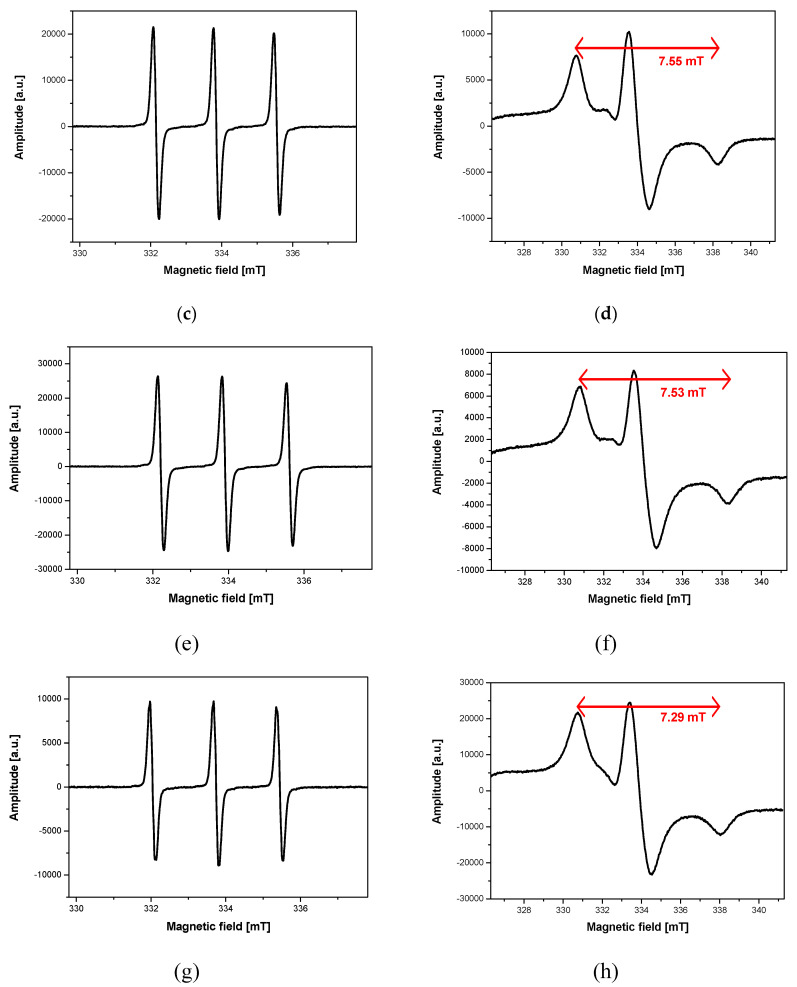
ESR spectra of the Fe_3_O_4_@SiO_2_@SiNHDOX@Dextran-TEMPOL nanoparticles: (**a**) aqueous solution of the nanoparticles recorded at 293 K in a magnetic field sweep range of 8 mT; (**b**) aqueous solution of the nanoparticles recorded at 240 K in a magnetic field sweep range of 15 mT; (**c**) the nanoparticles with MDA-MB-231 breast cancer cells recorded at 293 K in a magnetic field sweep range of 8 mT; (**d**) the nanoparticles with MDA-MB-231 breast cancer cells recorded at 240 K in a magnetic field sweep range of 15 mT; (**e**) the nanoparticles with HMEC recorded at 293 K in a magnetic field sweep range of 8 mT; (**f**) the nanoparticles with HMEC recorded at 240 K in a magnetic field sweep range of 15 mT; (**g**) the nanoparticles with yeast cells recorded at 293 K in a magnetic field sweep range of 8 mT; (**h**) the nanoparticles with yeast cells recorded at 240 K in a magnetic field sweep range of 15 mT.

The ESR spectrum of the nanoparticles with attached spin labels had the form shown in Figure 2 and Figure 3a. Figure 2 illustrates a typical ESR spectrum in the whole range of magnetic field. The broad line (ΔH = 84mT, g = 2.220) is derived from magnetite core with superparamagnetic properties, while a signal in the middle of the spectrum (enlarged in Figure 3a) is typical of a spin label.

The ESR spectra of the nanoparticles studied in suspensions without (Figure 3a) and with (Figure 3c) cells are characterized by similar structure and spectroscopic parameters, such as peak-to-peak line width, hyperfine splitting, and spectroscopic splitting factor. At room temperature (293 K), the molecular dynamics of magnetite core and spin labels are very high in the selected concentration range. Therefore, interaction anisotropy of the nanoparticles with the environment is largely averaged, and the differences in the values of spectroscopic parameters are within the limits of measurement uncertainty. In our previous studies [38], we clearly demonstrated that ESR spectra differentiated in lower temperatures of ESR measurements depending on environmental conditions. The differences were observed for the samples in various environments, including nanoparticles inside and outside cells. Finally, the measurement temperature of 240 K was chosen as an optimal value for ESR spectra recording of samples with cells incubated previously as standard at 310 K, because it guaranteed the greatest changes in the structure and spectroscopic parameters. In the selected conditions, the interactions of nanoparticles in a different environment, especially in the presence of cells, varied significantly (Figure 3b,d).

Figure 3 clearly demonstrates that ESR spectra of the nanoparticles studied without (Figure 3a,e,g) and with cancer cells (Figure 3c) are very similar in room temperature. At the same time, those recorded at 240 K are significantly different (Figure 3b,d). The differences observed are an effect of interaction and/or attachment of the nanoparticles to the cancer cells.

The differentiation of ESR spectrum structure recorded at 240 K was observed in the presence of cancer cells (Figure 3d), as well as human microvascular endothelial cells (Figure 3f) and yeast cells (Figure 3h) incubated previously at 310 K. Therefore, yeast cells were used as models for the analysis of interactions between nanoparticles and cells, including endocytosis and determination of changes in ESR spectrum parameters caused by this process. High-speed (almost immediate) nanoparticle–cell interaction, resulting in a wide triplet (ΔH = 7.55 mT, 7.53 mT, 7.29 mT; Figure 3d,f,h) presence in the ESR spectrum, was observed for all cells studied. According to the literature reports, endocytosis is divided depending on the process rate. Rapid endocytosis refers to the processes taking place in milliseconds, which distinguishes it from those much slower in time [37]. Thus, in our opinion, the spectrum in Figure 3d refers to the nanoparticles attached to a cell membrane. It results from very long correlation time at 240 K (τ = 10^−7^ s) and no interaction between the nanoparticles (see Figure 3b,d for comparison).

### 2.2. Effect of Incubation Time

**Figure 4:**
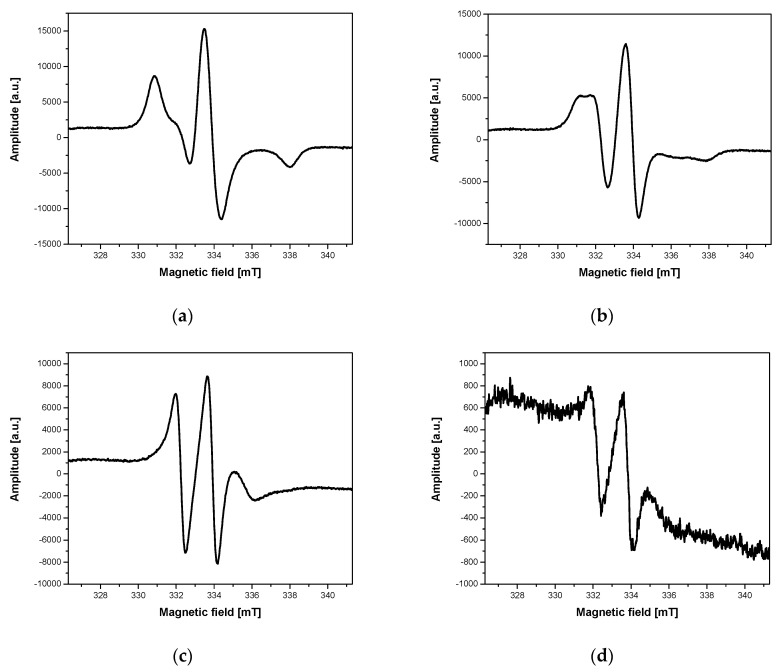
An example of changes in ESR spectra of Fe_3_O_4_@SiO_2_@SiNHDOX@Dextran-TEMPOL recorded at 240 K, depending on incubation time of the nanoparticle solution with yeast cells: (**a**) the beginning of incubation, (**b**) after 30 min of incubation, (**c**) after 90 min of incubation, (**d**) after 180 min of incubation.

Another important factor influencing nanoparticle–cell interaction is incubation time of cells with the nanoparticles. The examples of such temporal changes in ESR spectra are shown in Figure 4.

**Figure 5:**
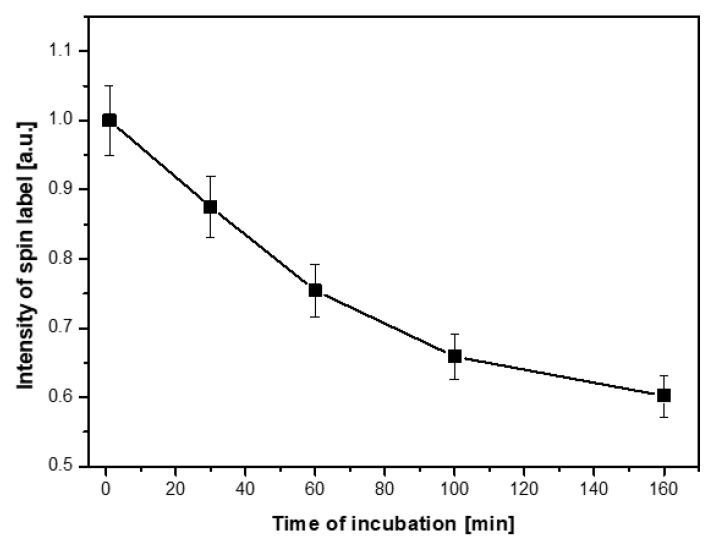
Changes in ESR signal intensity of Fe_3_O_4_@SiO_2_@SiNHDOX@Dextran-TEMPOL nanoparticles with yeast cells depending on incubation time.

To thoroughly examine the mechanisms responsible for changes in the structure of ESR spectra related to incubation time of nanoparticle solutions with cells, several experiments with different concentrations of nanoparticles/cells and different incubation times at 310 K were carried out. Figure 4 shows how the ESR spectra of the nanoparticle solution with cells change over time in terms of their structure and intensity. An exemplary result of changes in the ESR spectrum intensity in time is presented in Figure 5.

**Figure 6:**
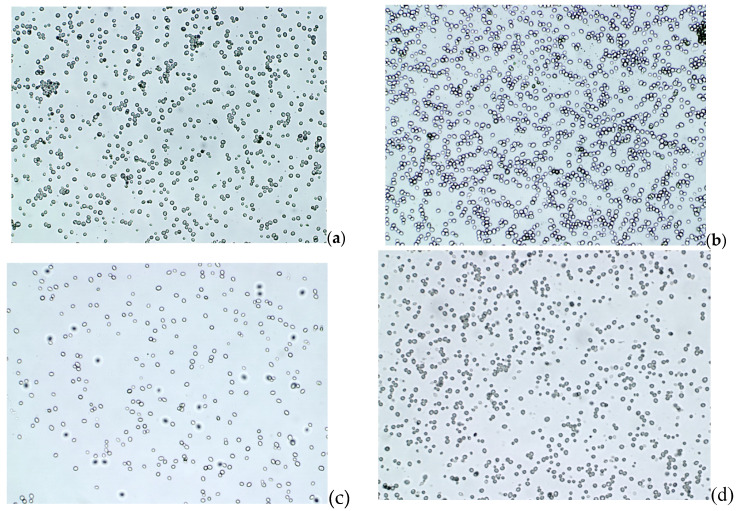
Microscopic photos of yeast cells in a solution with Fe_3_O_4_@SiO_2_@SiNHDOX@Dextran-TEMPOL nanoparticles: (**a**) the beginning of incubation, (**b**) after 3 h of incubation, and yeast cells without nanoparticles: (**c**) the beginning of incubation, (**d**) after 3 h of incubation.

The intensity of the spin label (Figure 4a–d and Figure 5) attached to the magnetic nanoparticles decreased as the incubation time increased, and it reached a value close to zero after a few hours. The rate of signal loss depends on the nanoparticle concentration, the number of cells in the incubated sample, and the incubation conditions. On the other hand, one would suppose that the decrease of the ESR spectrum intensity could be caused by the reduced number of cells in a solution. Therefore, microscopic photos (Figure 6) were taken to exclude such a reason for the observed changes. After analysis of a series of pictures taken, it was found that the number of cells increased with the incubation time. For example, the number of cells in yeast culture after 3 h of mixing them with the nanoparticles was approximately 2.4 times higher than initially while incubated without nanoparticles 2.6 (Table 5). These values do not differ significantly. Additionally, this is another confirmation that the nanoparticles studied do not exhibit cytotoxicity on yeast cells. This clearly shows that the decrease in ESR signal intensity (Figure 5) is due to the recombination of radicals and not to the decrease in cell number.

**Figure 7:**
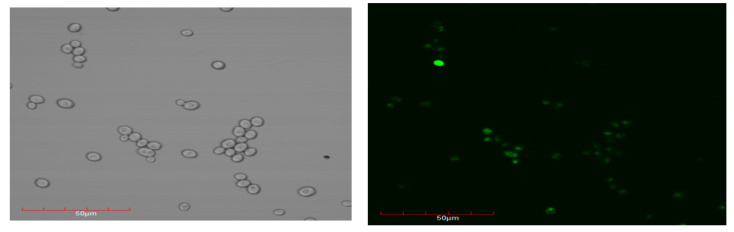
Confocal microscope images showing the entry of Fe_3_O_4_@SiO_2_@FITC@Dextran-TEMPOL nanoparticles into yeast cells.

In the next step, the penetration of the functionalized nanoparticles into the interior of yeast cells was investigated. A series of confocal microscope images were taken (Figure 7) using the fluorescein-labeled nanoparticles, Fe_3_O_4_@SiO_2_@FITC@Dextran-TEMPOL. The pictures confirmed the presence of the nanoparticles inside yeast cells.

**Figure 8:**
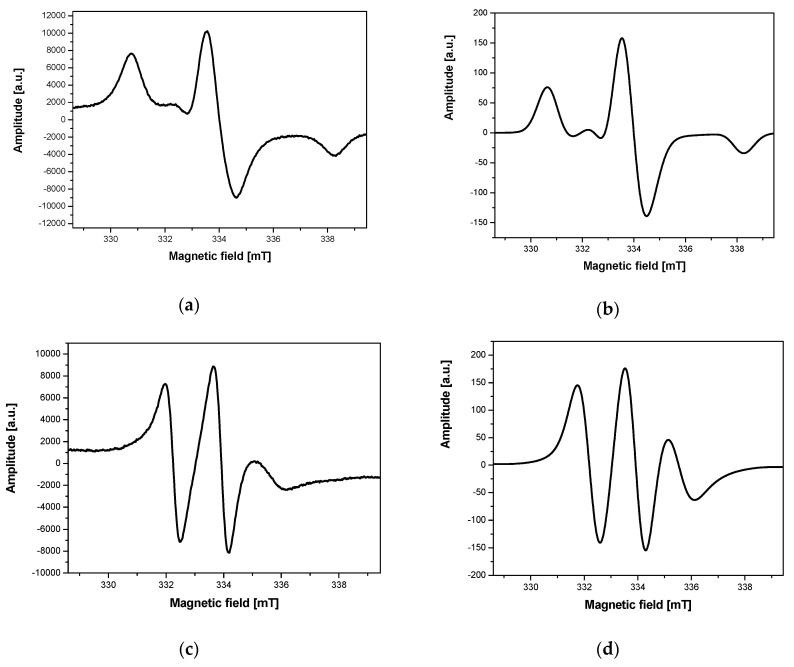
ESR spectra of TEMPOL attached to Fe_3_O_4_@SiO_2_@SiNHDOX@Dextran-TEMPOL: (**a**) a so-called broad experimental triplet, (**b**) a broad simulated triplet, (**c**) a narrow experimental triplet, (**d**) a narrow simulated triplet.

The incubation time influenced not only the ESR spectrum intensity but also its structure. Initially, the nanoparticles with the attached spin label interacted with cells (especially with a cell membrane) and the ESR spectrum recorded at 240 K was characterized by the presence of a wide triplet with a larger or smaller participation of a narrow triplet interpreted as the presence of the nanoparticles inside cells (Figure 3d and Figure 4a). As the incubation continued (at 310 K), the narrow triplet (Figure 4b) gradually dominated until the ESR spectrum was fully converted to the narrow triplet (Figure 4c). The rate of these changes strongly depends on the nanoparticle concentration, the number of cells, their type, and viability. The measurements taken at a temperature preventing endocytosis (274 K), using cooled solutions of the nanoparticles and cells, give ESR spectra with a wide triplet, and a narrow triplet does not appear over time. The ESR spectrum is similar, then, to that shown in Figure 8a. Increasing the incubation temperature from 274 K to 310 K, when the endocytosis process was efficient, the narrow triplet was observed in the ESR spectrum (Figure 8c).

On the other hand, if the sample is filtered (0.45 µm), no spin label signals are observed in the filtrate. However, it is visible in the material left on the filter. It proves that the narrow triplet comes from cell-associated spin labels, not from molecules in extracellular solution.

The dynamics and structure of the ESR spectra were changed during the experiments. Therefore, the EasySpin simulation program was used to determine several parameters, including spin label correlation times. Figure 8 shows the results of the spectrum simulation with the so-called wide triplet (Figure 8a,b) and narrow triplet (Figure 8c,d). The simulations were carried out for the following spin label spectroscopic parameters: g factor (2.0027, 2.0069, 2. 0085), hyperfine splitting A (0.67, 0.65, 3.86 mT), and correlation time τ = 10^−7^ s (Figure 8b), τ = 10^−9^ s (Figure 8d).

### 2.3. Modeling of Endocytosis Process

**Figure 9:**
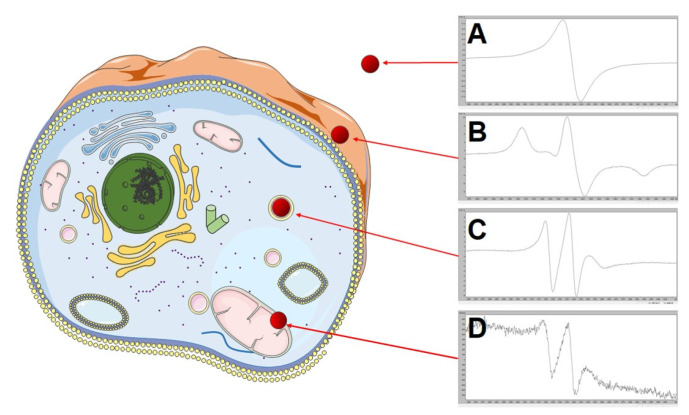
Changes in the structure and intensity of ESR spectra in the endocytosis process of the functionalized nanoparticles in a cell: A—ESR signal from the spin label not bonded to cells, B—ESR signal from the spin label attached to a cell, C—ESR signal from the spin label located inside cells (in organelles such as endosome or lysosome), D—ESR signal from the spin label probably present in cellular mitochondria. This figure was created using Servier Medical Art templates, which are licensed under a Creative Commons Attribution 3.0 Unported License; https://smart.servier.com.

The interaction process of the nanoparticles studied with cells can be divided into three main steps. The first one corresponds to the beginning of interacting nanoparticles–cells. In this early step, the ESR spectrum of a free radical 4-Hydroxy-TEMPO (TEMPOL) recorded at 240 K is characterized by a single line, similar in shape and parameters to that of the nanoparticle solution without cells (Figure 3b and Figure 9A). The second step is characterized by the differentiation of the ESR spectrum to a wide triplet (Figure 8a and Figure 9B) and extended correlation time (τ = 10^−7^ s). The third (last) step is characterized by the presence of a narrow triplet (Figure 8c and Figure 9C) and a short correlation time (τ = 10^−9^ s).

The entire process of changes in the structure and intensity of ESR spectrum during incubation of the nanoparticles with cells can be interpreted as follows. After mixing the nanoparticle solution with cells at a temperature that inhibits endocytosis (273 K), the magnetic core and the spin labels interact strongly with each other and do not bind to cells. It results in the presence of a single narrow line in the ESR spectrum recorded at 240 K (Figure 9A). After a certain incubation period at 310 K, the nanoparticles attach to a cell—to be more precise, probably to a cell membrane. A wide triplet reflects this as an effect of longer correlation time and reduced interactions between spin labels (Figure 8a and Figure 9B). In the third step, the nanoparticles are located inside cells (as shown in Figure 1F–G), presumably in organelles such as endosome/lysosome, however, further in vitro testing has to be done. It promotes rapid molecular movements and short correlation time at 240 K, and the spectrum is in the form of a narrow triplet (Figure 8c and Figure 9C). As the incubation time increases, the recombination of spin labels begins as a result of reactions with radicals, reactive oxygen species, and so forth. These reactions probably occur in cellular mitochondria. After several dozen minutes to several hours, the concentration of spin labels in a solution decreases several times (Figure 9D), until there is a complete disappearance of spin label radicals. The period depends on the spin label type and coverage of the magnetic core.

Both the shape of the ESR spectrum (narrow triplet) and the short correlation time (τ = 10^−9^ s) testify to the high dynamics of movement of the TEMPOL spin label attached to magnetic nanoparticles. This situation is possible only inside the cell because at 240 K (ESR spectra recording temperature), the external environment is frozen. Thus, these results confirm the presence of nanoparticles in the interior of the cell and the usefulness of the ESR method for monitoring the process of endocytosis in cells.

Modeling of the endocytosis process of the nanoparticles having spin labels is very complex. We present here our attempt to model it. In the second step of the endocytosis process—penetration of nanoparticles into the cell—differences in nanoparticle concentration outside and inside cells can be approximated by Equations (1) and (2). The changes in concentration during incubation of cells with nanoparticles in the extracellular environment, A_ext_, caused by entering of nanoparticles inside the cell will be described by Equation (1):

A_ext_ = 1 − exp(−(i−1)/160) (1)

while those inside the cell, A_int_, by Equation (2):

A_int_ = exp(−(i−1)/160) (2)

**Figure 10:**
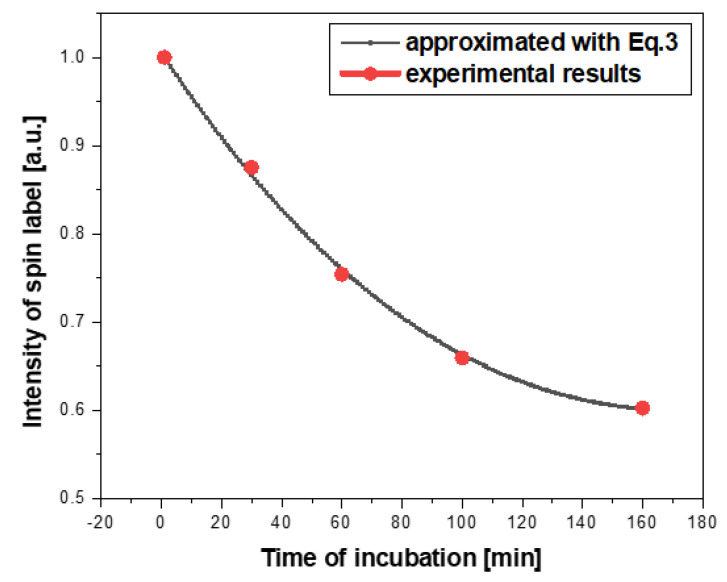
Changes in the intensity of the spin label attached to the magnetic nanoparticles during incubation with cells. The approximation was made according to Equation (3).

The total concentration of spin label should be constant over time in the absence of spin label recombination. According to experimental data (Figure 5), the intensity of the ESR spin label signal decreases rapidly during incubation. It proves the participation of spin labels attached to the nanoparticles in redox reactions occurring most likely in cellular mitochondria. The whole process of ESR spectrum intensity changes can be approximated using Equation (3). The following assumptions were made: exponential decrease in intensity as a result of spin label radical recombination, a small correction on recombination rate during incubation as a result of a change in the number of cells in solution, and a decrease in recombination efficiency (Figure 10).

A_tot_ = A_ext_ + A_int_ ∗ exp(−(t)/a) ∗ b + c ∗ t^2^ (3)

where A_tot_ is total intensity of ESR spin label, b = 0.17, a = 500, c = 6 ∗ 10^−6^ are fixed coefficients, and t is time.

**Figure 11:**
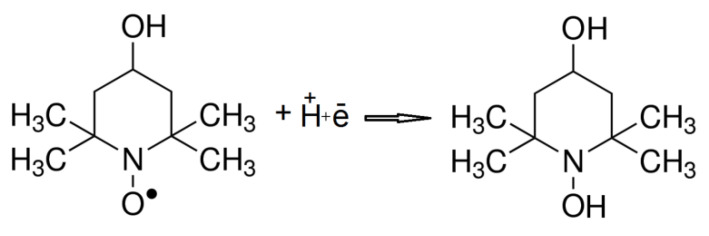
The recombination of TEMPOL spin label.

Recombination of spin labels attached to a magnetite core proceeds by connecting protons to a spin label radical (Figure 11). However, a detailed description and location of such reactions require further studies. The process probably occurs in cellular mitochondria as an effect of aerobic respiration, intense enzymatic responses, and generation of reactive oxygen species (ROS).

## 3. Materials and Methods

### 3.1. Materials

FeCl_3_·6H_2_O and FeCl_2_·4H_2_O, hexamethylene diisocyanate, 3-isocyanatopropyltriethoxysilane, tetraethyl orthosilicate (TEOS), 4-hydroxy-TEMPO (TEMPOL), doxorubicin hydrochloride (DOX), fluorescein isothiocyanate (FITC), and dextran and solvents were purchased from Sigma-Aldrich (Poznań, Poland). Other chemicals were the analytic grade reagents commercially available and used without further purification. Aqueous solutions were prepared with distilled water.

### 3.2. Synthesis Procedures

The nanoparticles studied were obtained and characterized using the processes and methods previously described [38]. To understand the structures of functionalized nanoparticles, the short description of synthesis procedures are given below.

#### 3.2.1. Synthesis of Fe_3_O_4_@SiO_2_@SiNHDOX@Dextran-TEMPOL

The mixture containing FeCl_2_ and FeCl_3_ was stirred under a nitrogen atmosphere. After addition of ammonia and sonication, the mixture was heated and the product, Fe_3_O_4_, was magnetically separated. In the next process, magnetite nanoparticles were covered with a silica layer. Magnetic silica nanoparticles, Fe_3_O_4_@SiO_2_, were prepared according to the Ströber method, using tetraethyl orthosilicate (TEOS) dissolved in ethanol. Next, Fe_3_O_4_@SiO_2_ was used to obtain Fe_3_O_4_@SiO_2_@SiNHDOX. For this purpose, (3-isocyanatopropyl) triethoxysilane in dimethyl sulfoxide (DMSO) was placed in a flask, and then doxorubicin hydrochloride and triethylamine in DMSO were added. The mixture was stirred, and then the crude product was added to the portion of Fe_3_O_4_@SiO_2_ magnetic particle solution. The resulting mixture was stirred at room temperature. The product, Fe_3_O_4_@SiO_2_@SiNHDOX, was magnetically collected, washed, and dried. Finally, it was covered with dextran functionalized by 4-Hydroxy-TEMPO (Dextran-TEMPOL) and a stable product Fe_3_O_4_@SiO_2_@SiNHDOX@Dextran-TEMPOL in water was obtained.

#### 3.2.2. Synthesis of Fe_3_O_4_@SiO_2_@FITC-Dextran-TEMPOL

The surface of silica-coated magnetic particles (Fe_3_O_4_@SiO_2_) was functionalized with a fluorescent organic dye (FITC). The Fe_3_O_4_@SiO_2_ was dispersed in acetone, followed by the addition of fluorescein isothiocyanate (FITC). The product Fe_3_O_4_@SiO_2_@FITC was separated by a magnet, washed, and dried. Then, the magnetic particles were covered with Dextran-TEMPOL to obtain a stable product Fe_3_O_4_@SiO_2_@FITC@Dextran-TEMPOL in water.

**Figure 12:**
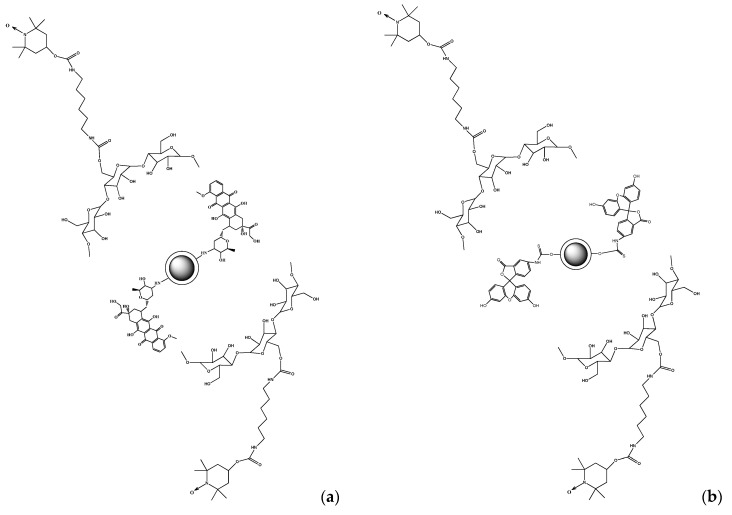
The structures of Fe_3_O_4_@SiO_2_@SiNHDOX@Dextran-TEMPOL (**a**) and Fe_3_O_4_@SiO_2_@FITC@Dextran-TEMPOL (**b**) nanoparticles.

The structures of Fe_3_O_4_@SiO_2_@SiNHDOX@Dextran-TEMPOL and Fe_3_O_4_@SiO_2_@FITC@Dextran-TEMPOL are presented in Figure 12.

### 3.3. Procedures with Human Microvascular Endothelial, Breast Cancer, and Yeast Cells

Endocytosis was studied using human microvascular endothelial cells (HMEC), breast cancer cells (obtained from cell culture conducted at Faculty of Biology, Adam Mickiewicz University in Poznań), and yeast cells.

Human microvascular endothelial cells (HMEC) [39] were provided by the Center for Disease Control and Prevention (Atlanta, GA, USA) and cultured in MCDB-131 medium (IITD PAN, Wroclaw, Poland) containing 10% of fetal bovine serum (FBS) (Gibco, Thermo Fisher Scientific, Waltham, MA USA), 100 U/mL penicillin, 100 μg/mL streptomycin, 0.01 μg/mL EGF, 0.05 μM hydrocortisone, and 2 mM L-glutamine. The MDA-MB-231 breast cancer cells [40] were cultured in DMEM medium (IITD PAN, Wroclaw, Poland) containing 10% of fetal bovine serum (FBS) (Gibco, Thermo Fisher Scientific), 100 U/mL penicillin, and 100 μg/mL streptomycin, 0.01 μg/mL.

For assessment of cytotoxic effects of the nanoparticles on cells, the EZ4U - Cell Proliferation and Cytotoxicity Assay (Biomedica Medizinprodukte, Vienna, Austria) was used according to the manufacturer’s protocol. Fluorescent nanoparticles, Fe_3_O_4_@SiO_2_@FITC@Dextran-TEMPOL, were added to HMEC in concentrations up to 150µg/mL and incubated for 24 h, before observing them under a Nikon A1 confocal microscope system at 100x magnification.

Bakery yeast (Saccharomyces cerevisiae) (1 g) was diluted in 50 mL of distilled water with the addition of 0.3 g of sugar and incubated at 310 K for 1 h. To check if the presence of nanoparticles has an impact on yeast cell proliferation, for each sample, five microscopic pictures were taken. The number of cells was calculated using Image J, Java-based image processing program. The yeast cell growth after 3 h incubation was then determined for the sample with and without nanoparticles.

Water solutions of the functionalized magnetite nanoparticles were mixed with each cell sample and incubated during a few hours as standard (310 K). They were studied at specified time intervals using the ESR method.

### 3.4. ESR Measurements

For electron spin resonance (ESR) measurements, an X-band Bruker EMX-10 spectrometer was used with a magnetic field second modulation frequency of 100 kHz. After incubation at 310 K, the samples were taken to Pasteur pipettes and measured at 293 K and 240 K. Measurement temperatures were maintained and controlled by a Bruker temperature controller unit ER 4131VT. The ESR spectra were recorded in three magnetic field ranges: 650 mT, 15 mT, and 8 mT.

For registered ESR spectra, typical spectroscopic parameters were determined: g-spectroscopic splitting factor value, peak-to-peak line width (ΔH), and hyperfine splitting constant (A) [22,38] with the accuracy of ± 0.0005, ± 0.5 mT, and ± 0.5 mT, respectively. Each time, the concentration of spin label was calculated from the integrated intensity of appropriate ESR signals with the accuracy of 10%. All ESR experiments were repeated many times giving the same ESR spectra.

### 3.5. Simulations in EasySpin

ESR spectra were simulated and fitted in EasySpin 5.2.15, a MATLAB toolbox. For a simulation of each type of spectrum, this software uses a specific function based on the algorithms that determine the accuracy and the speed of the simulation. There are three primary functions: chili, garlic, and pepper.

In this study, the chili function was used as suitable for slow-moving molecules with one or more nuclei in a weak interaction with the unpaired electron. This function is defined by two input arguments: static and dynamic parameters of the circuit spin, g-spectroscopic splitting factor value (g), peak-to-peak line width (ΔH), spin (S), hyperfine splitting constant (A), and correlation time (τ). For simulation, experimental parameters are required: central field, magnetic field sweep range, and frequency. The esfit function allows adjusting the experimental spectrum to the data important for the suitable type of molecule. It also allows specifying simulated parameters, which correspond to the ESR spectrum in the best way [20,41].

### 3.6. Statistical Analysis

EZ4U cytotoxic assay, for assessment of cytotoxic effects of the nanoparticles on cells, was performed in three replicates. One-way ANOVA with Bonferroni post-test was performed to test for statistical significance. The results were presented as mean ± standard deviation (SD).

In the case of yeast cell proliferation with and without nanoparticles, data were shown as mean values ± standard deviation (SD). Statistical significance was defined by one-way ANOVA analysis and the significance was defined as *p* < 0.05.

## 4. Conclusions

ESR spectroscopy enables studying of the endocytosis process of the nanoparticles with attached spin labels in different cells (i.e., cancer, endothelium, and yeast cells). It is an appropriate method for direct monitoring of free radical reactions occurring inside cell organelles, involving nanoparticles. Moreover, ESR spectroscopy allows evaluating in vitro effects of cell viability, concentration, and biocompatibility of nanoparticles on the efficiency of endocytosis processes.

## Figures and Tables

**Table 1:** Cell viability as percentage of control for nanoparticles with TEMPO spin label.

Replicate	Control	005 µM	01 µM	05 µM	1 µM	5 µM	25 µM
1st	100.0000	90.916570	92.000170	72.982670	76.579710	67.436440	63.858640
2nd	100.0000	90.628040	88.633960	74.951110	85.383160	73.950870	78.958500
3rd	100.0000	95.475390	91.718050	77.919780	89.916330	80.580700	77.092660

**Table 2:** Cell viability as percentage of control for nanoparticles with TEMPO spin label and FITC.

Replicate	Control	0.05%	0.1%	0.5%	1%	5%	10%	25%	50%
1st	100.0000	80.381940	96.283270	91.974530	90.006090	93.295360	86.575760	90.570340	53.766420
2nd	100.0000	91.685990	84.241850	82.004130	81.734830	74.053450	86.043580	68.443100	79.279100
3rd	100.0000	74.527930	84.171330	73.816220	93.276120	88.159480	81.318060	85.716580	76.740010

**Table 3:** Statistical analysis for results collected in Table 1. Asterisks represent degree of significance (* = *P* < 0.05; *** = *P* < 0.001).

Bonferroni’s Multiple Comparison Test	Mean Diff.	t	Significant? *p* < 0.05?	Summary	95% CI of diff
Control vs. 05 µM	24.72	6.071	Yes	***	9.656 to 39.77
Control vs. 1 µM	16.04	3.940	Yes	*	0.9810 to 31.10
Control vs. 5 µM	26.01	6.389	Yes	***	10.95 to 41.07
Control vs. 25 µM	26.70	6.558	Yes	***	11.64 to 41.76

**Table 4:** Statistical analysis for results collected in Table 2. Asterisk represents degree of significance (* = *P* < 0.05).

Bonferroni’s Multiple Comparison Test	Mean Diff.	t	Significant? *p* < 0.05?	Summary	95% CI of diff
Control vs. 50%	30.07	4.227	Yes	*	3.222 to 56.92

**Table 5:** Statistical analysis of yeast cells growth after 3 h incubation without and with nanoparticles.

Replicate	Yeast Cells with Nanoparticles Growth	Yeast Cells without Nanoparticles Growth
1	2.4	2.6
2	2.5	2.6
3	2.4	2.7
4	2.4	2.3
5	2.3	2.8
Mean	2.4	2.6
SD	0.07	0.19
SE	0.03	0.08

## Abbreviations

ESR	Electron spin resonance
TEMPOL	4-hydroxy-TEMPO spin label
HMEC	Human microvascular endothelial cells
TEOS	Tetraethyl orthosilate
DOX	Doxorubicin hydrochloride
FITC	Fluorescein isothiocyanate
